# Experimental data on synthesis and characterization of WO_3_/TiO_2_ as catalyst

**DOI:** 10.1016/j.dib.2019.104151

**Published:** 2019-06-15

**Authors:** Augusto Arce-Sarria, Cindy Lorena Caicedo-Rosero, Jose Antonio Lara-Ramos, Jennyfer Diaz-Angulo, Fiderman Machuca-Martínez

**Affiliations:** Escuela de Ingeniería Química, Universidad del Valle, A.A. 23360, Cali, Colombia

**Keywords:** WO_3_/TiO_2_ compound, Prevents recombination, Sol-gel method

## Abstract

WO_3_/TiO_2_ is a composite photocatalyst that is being widely used in heterogeneous photocatalysis because it presents better photocatalytic properties than TiO_2_. For example, the probability of recombination of the electron/hole pairs is diminished and a more range of the solar spectrum is used for its excitation. However, this depend of variables such as tungsten oxide concentration, calcination temperature and synthesis method. This work is focused in establish the effect of WO_3_ on the morphological and structural characteristics of TiO_2_. WO_3_/TiO_2_ was synthesized by sol-gel method at different calcination temperatures and at different concentrations of tungsten oxide. The surface area, the possible transition between valence band and conduction band, particle size, elemental analysis and crystallography were examined through the BET, DRS, SEM-EDS and XRD analysis.

**Table:** Specifications table

Subject area	Chemical engineering, chemistry
More specific subject are	Synthesis and characterization of materials
Type of data	Figure and table
How data was acquired	Data were obtained by means of characterization techniques such as XRD, SEM-EDS. The specific surface area was determined by BET methodology and the band gap (Eg) values were determined by Kubelka-Munk methodology from DRS analysis.
Data format	Analyzed
Experimental factors	Effect of the calcination temperature (500, 600 and 700 °C) and percentages by weight of WO_3_ (1, 3, 5% w/w) on the characteristics of the photocatalyst such as crystal structure and morphology were evaluated.
Experimental features	Synthesis of WO_3_/TiO_2_ was carried out through the implementation of the Sol-Gel method with the objective of improving the photocatalytic activity of TiO_2_ having as a response variable the reduction of Eg, which could imply a redshift in terms energy absorption that result beneficial in photocatalytic process under natural sunlight.
Data source location	GAOX, Universidad del Valle, Cali, Colombia.
Data accessibility	The data is found only in this article. A. Arce-Sarria, F. Machuca-Martínez, C. Bustillo-Lecompte, A. Hernández-Ramirez, and J. Colina-Márquez, Degradation and Loss of Antibacterial Activity of Commercial Amoxicillin with TiO_2_/WO_3_-Assisted Solar Photocatalysis, Catalysts. 8 (2018) 1–14. https://doi.org/10.3390/catal8060222[1].

**Table:** 

**Value of data** • Data obtained allow knowing the calcination temperature effect and the percentage by weight of WO3 in the crystalline structure of the synthesized photocatalyst. • It can be observed that the addition of WO3 allows that anatase phase of TiO2 be more thermally stable which could contribute to the improvement of photocatalytic activity of WO3/TiO2. • The bang gap data for each sample of photocatalyst at different percentage by weight of WO3 and calcination temperature were obtained, which could serve as references for improving the doping with another oxide. • Data may be useful for future research.

## 1 Data

Doping TiO_2_ pretend to improve its photocatalytic performance, since even though it presents a great effectiveness in the degradation of recalcitrant compounds only it achieves its excite state by absorption UV energy, which correspond to 5% of solar spectrum. So more than 50% of visible radiation is being wasted [2], [3]. Therefore, it is necessary the coupling of this catalyst with another compound or mixed oxides and characterize the properties of the new materials product of doping. In this case WO_3_/TiO_2_. Some physicochemical properties of titanium oxide and tungsten oxide are shown in Table 1.

**Table 1:** Physicochemical properties of oxides.

	Titanium Oxide	Tungsten Oxide
Molecular structure		
Molecular formula	TiO_2_	WO_3_
Molecular Weight [g/mol]	79.8	231.8
Band gap Eg [eV]	3.2	2.8
Water solubility	Insoluble	Insoluble

## 2 Experimental design, materials and methods

Calcination temperature directly affects the crystalline structure of TiO_2_. It was found that anatase phase presents a better photocatalytic performance than rutile phase [4], so in this work three calcination temperatures 500, 600 and 700 °C were evaluated. Another parameter for improving the photocatalytic activity of TiO_2_ is the doping percentage by weight of WO_3_, which favor the shift in the energy absorption toward visible light region. In this case it was varied in 1, 3 and 5% w/w.

### 2.1 General procedure

The synthesis of WO_3_/TiO_2_ photocatalyst was carried out by Sol-Gel methodology using Titanium (IV) n-butoxide, 98+ % ACROS (CAS RN 5593-70-4) and p-Tungstate ammonium, 99.99% Aldrich (CAS 11120-25-5) as precursors of the obtained materials [5].

### 2.2 Characterization

Surface area of the photocatalyst obtained was determined by nitrogen physisorption onto material surface using the Brunauer, Emmett and Teller (BET) theory. The Kubelka-Munk function was used for estimating the Eg based on the reflectance spectroscopy values [1]. The surface area and band gap of the synthesized photocatalyst to different conditions are shown in Table 2 and Table 3, respectively.

**Table 2:** Surface area of bare TiO_2_ and TiO_2_ doped with WO_3_ (m_2_/g).

	Calcination temperature		
WO_3_ percentage	500 °C	600 °C	700 °C
0%	41.8	5.4	0.11
1%	52.3	16.8	0.16
3%	64.6	37.2	10.2
5%	68.7	45.1	18.4

**Table 3:** Band gap results of WO_3_/TiO_2_ particles.

Sample	Eg (eV)	Wavelength (nm)
TiO_2_-500 °C	3.18	388.72
TiO_2_-600 °C	3.15	391.99
TiO_2_-700 °C	3.11	396.65
1% WO_3_/TiO_2_-500 °C	3.04	406.37
1% WO_3_/TiO_2_-600 °C	2.98	414.69
1% WO_3_/TiO_2_-700 °C	2.96	416.82
3% WO_3_/TiO_2_-500 °C	3.02	408.57
3% WO_3_/TiO_2_-600 °C	2.97	416.30
3% WO_3_/TiO_2_-700 °C	2.95	418.45
5% WO_3_/TiO_2_-500 °C	2.99	413.44
5% WO_3_/TiO_2_-600 °C	2.95	418.15
5% WO_3_/TiO_2_-700 °C	2.93	420.92

**Fig. 1:**
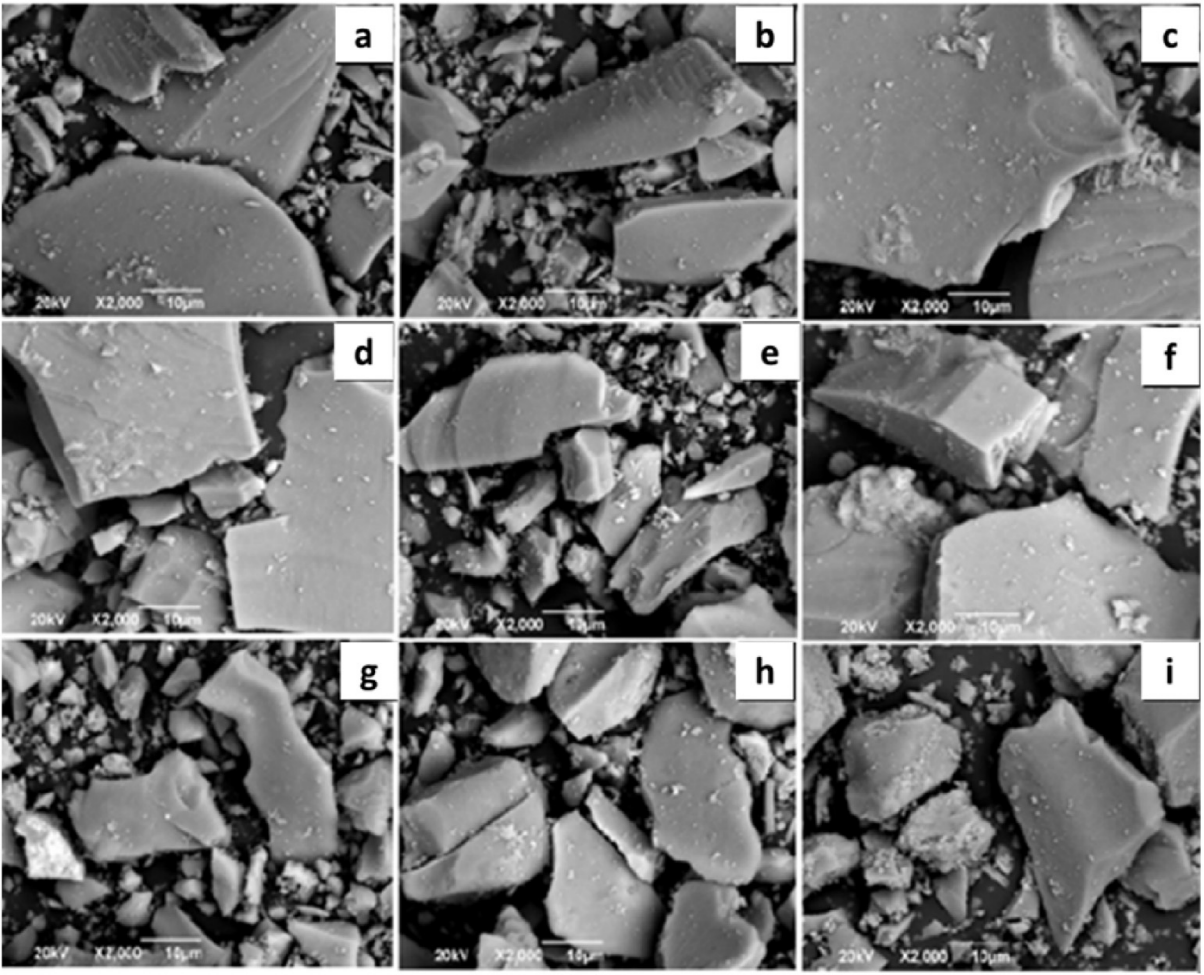
SEM for WO_3_/TiO_2_ materials. (a) TiO_2_-600 °C, (b). TiO_2_-700 °C, (c). 1% WO_3_/TiO_2_-600 °C, (d). 1% WO_3_/TiO_2_-700 °C, (e). 3% WO_3_/TiO_2_-600 °C, (f). 3% WO_3_/TiO_2_-700 °C, (g). 5% WO_3_/TiO_2_-500 °C, (h). 5% WO_3_/TiO_2_-600 °C, (i). 5% WO_3_/TiO_2_-700 °C.

In order to know the morphology and composition WO_3_/TiO_2_ photocatalyst samples, SEM and EDS analyzes were performed. The results are shown in Fig. 1 and Table 4 respectively.

**Table 4:** Elemental composition according to EDS analysis, given in percentage of element in the sample.

Sample	O	Ti	W
TiO_2_ - 600 °C	43.27	56.73	–
TiO_2_ - 700 °C	47.28	52.72	–
1% WO_3_/TiO_2_ - 600 °C	44.29	54.82	0.89
1% WO_3_/TiO_2_ - 700 °C	43.07	56.05	0.88
3% WO_3_/TiO_2_ - 600 °C	43.09	54.27	2.64
3% WO_3_/TiO_2_ - 700 °C	38.76	58.54	2.71
5% WO_3_/TiO_2_ - 500 °C	51.08	44.08	4.84
5% WO_3_/TiO_2_ - 600 °C	41.91	52.95	5.14
5% WO_3_/TiO_2_ - 700 °C	36.77	58.46	4.78

**Fig. 2:**
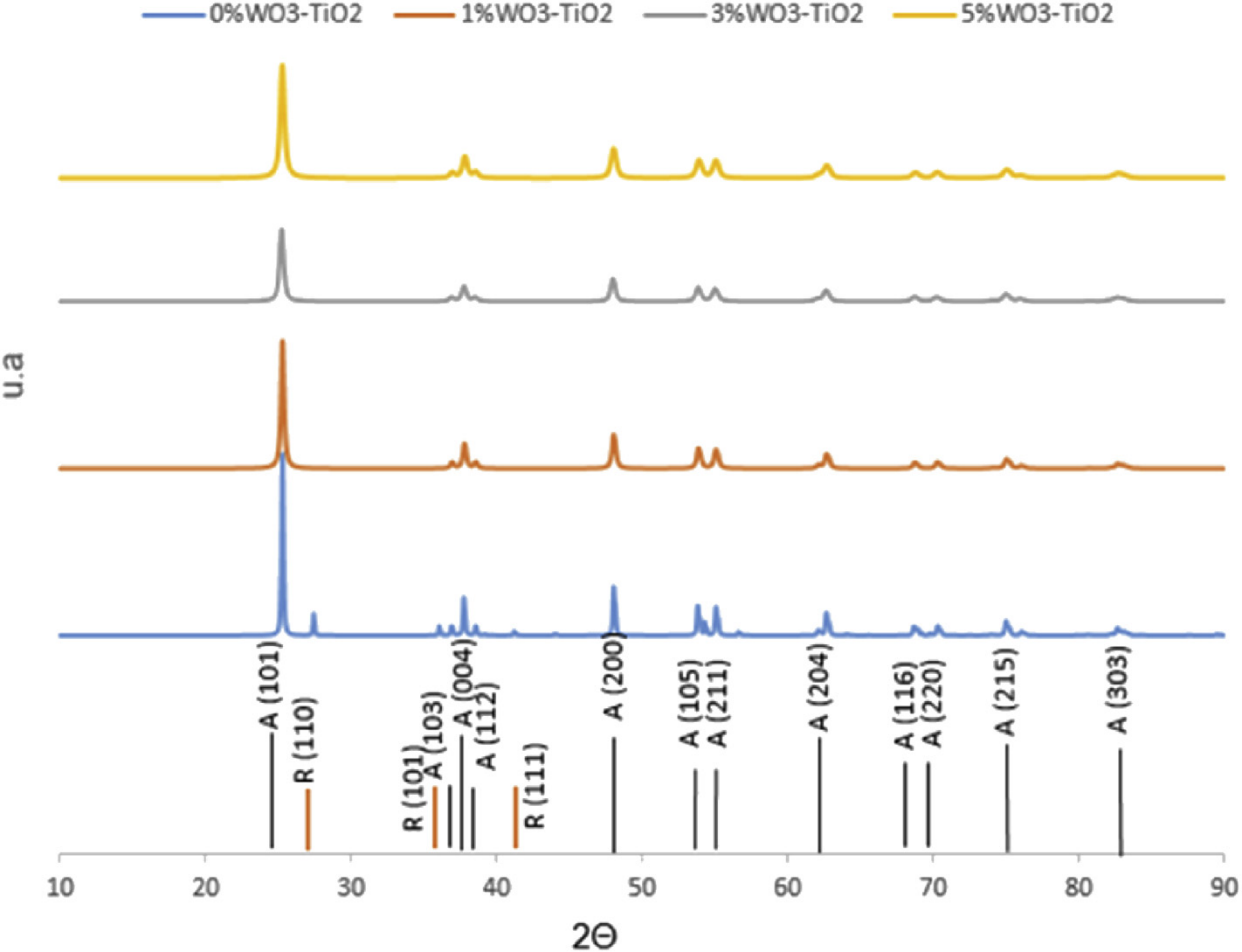
Difractograms obtained from samples synthesized from WO_3_/TiO_2_ calcined at 600 °C (Anatase) and R (Rutile).

**Fig. 3:**
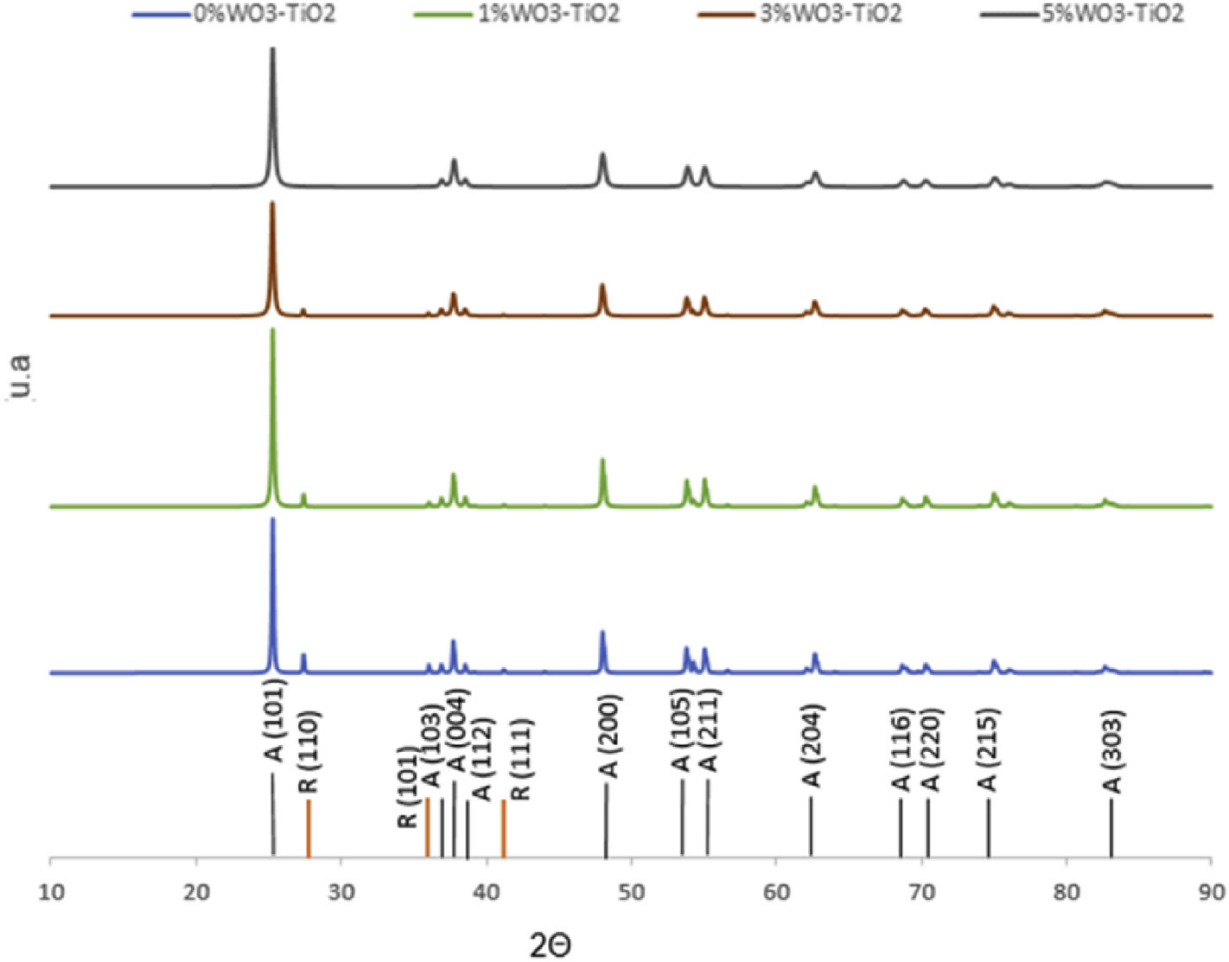
Difractograms obtained from samples synthesized from WO_3_/TiO_2_ calcined at 700 °C A (Anatase) and R (Rutile).

XRD analysis was performed on samples calcined at 600 °C (Fig. 2.) and 700 °C (Fig. 3.) because at these temperatures the crystalline transition is achieved. JCPDS 21–1272 and JCPDS 21–1276 cards were used as patterns for the anatase phase and the rutile phase respectively.

**Fig. 4:**
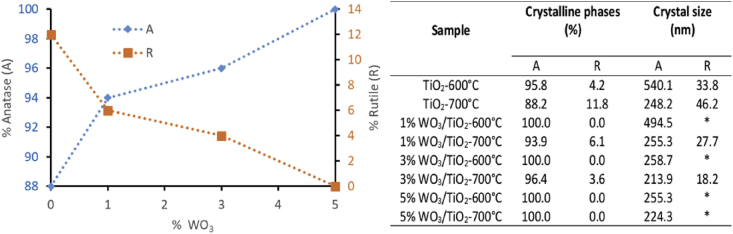
Diagram of crystalline phases distribution A (Anatase), R (Rutile) and crystal size.

The Fig. 4 shows the relationship between the Anatasa and Rutile phase on different catalysts synthesized.
